# Molecular evidence of *Plasmodium vivax* infection in Duffy negative symptomatic individuals from Dschang, West Cameroon

**DOI:** 10.1186/s12936-017-1722-2

**Published:** 2017-02-14

**Authors:** Gianluca Russo, Giovanni Faggioni, Giacomo Maria Paganotti, Ghyslaine Bruna Djeunang Dongho, Alice Pomponi, Riccardo De Santis, Gianpiero Tebano, Mpoame Mbida, Martin Sanou Sobze, Vincenzo Vullo, Giovanni Rezza, Florigio Romano Lista

**Affiliations:** 1 Department of Public Health and Infectious Diseases, Sapienza University of Rome, Piazzale Aldo Moro, 00185 Rome, Italy; 2 Department of Molecular Biology, Immunology and Experimental Medicine, Army Medical and Veterinary Research Centre, Via di Santo Stefano Rotondo, 00184 Rome, Italy; 3 BUP Core Laboratory, Botswana-University of Pennsylvania Partnership (BUP), P O Box AC 157 ACH, Gaborone, Botswana; 4 Department of Biomedical Sciences, University of Dschang, BP 96 Dschang, Cameroon; 5 Department of Infectious, Parasitic and Immune-mediated Diseases, Istituto Superiore di Sanità, Viale Regina Elena, 299, 00161 Rome, Italy

**Keywords:** Cameroon, Duffy antigen genotype, Malaria, *Plasmodium vivax*

## Abstract

**Background:**

*Plasmodium vivax* infection is known to be rare in West/Central Africa, the most accepted explanation being the lack of expression of erythroid Duffy antigen in the local human populations. Duffy negativity prevents the parasite to exploit the entry mechanism on the red blood cell surface. However, there are a growing number of reported vivax infections in Duffy-negative individuals. Data on *P. vivax* circulation in Cameroon are limited. The aim of the study was to evaluate the *P. vivax* presence, and its association with the Duffy genotype in West Cameroon.

**Results:**

Overall, 484 blood samples were collected consecutively from febrile outpatients attending the Dschang’s Hospital (West Cameroon) during a 3-months period. *Plasmodium vivax* infection was detected by PCR in 5.6% (n = 27/484) of the cases, representing 38.6% (n = 27/70) of all *Plasmodium* infections detected. All *P. vivax* infected individuals showed a Duffy-negative genotype, and the frequency of Duffy-positive individuals in the whole tested population was 1.7%.

**Conclusions:**

The results of this study confirm the circulation of *P. vivax* in Cameroon, as well as that the lack of expression of Duffy-antigen does not confer full protection against vivax malaria acquisition.

## Background

The World Health Organization (WHO) has estimated that a total of 214 million new cases of malaria occurred globally in 2014, with 438,000 related deaths, mostly related to *Plasmodium falciparum* and located in sub-Saharan Africa [1]. *Plasmodium vivax* is responsible of 8% of estimated malaria cases worldwide (about 50% when excluding sub-Saharan Africa), with three countries (Ethiopia, India and Pakistan) accounting for 80% of the cases [1]. *Plasmodium vivax* has a wider geographic distribution than *P. falciparum,* which is mostly prevalent in sub-Saharan Africa [1]. Possible explanations for this could be that *P. vivax* is able to develop at lower temperatures in the vector [2, 3], and may survive for long periods as hypnozoite (dormant liver stage) representing a possible reservoir of the infection and a major obstacle toward vivax malaria eradication [4–6].

For a long time, scientists and decision makers have neglected malaria due to *P. vivax* because it was considered as “benign” malaria when compared with the “malignant” form caused by *P. falciparum* [2]. Clinical manifestations of *P. vivax* infection are generally less severe than those related to *P. falciparum* infection, possibly because *P. vivax* infects mainly young red cells (reticulocytes), which are numerically limited (being <2.5% of circulating red blood cells) and appear to be less prone to cytoadherence or sequestration in microcirculation [2, 7, 8]. Nevertheless there are still remarkably large knowledge gaps on the pathophysiology of vivax malaria [7–9].

The human Duffy antigen receptor is the only well-known pathway used by *P. vivax* for erythrocyte invasion, through the interaction with the *P. vivax* Duffy binding protein (PvDBP) [10]. The Duffy antigen, also called Duffy Antigen Receptor for Chemokines (DARC), is a multimeric membrane protein organized into 7 trans-membrane domains that is present in erythrocytes, endothelial cells (bone marrow, lung, kidney, adrenal gland, thyroid, spleen, colon), and epithelial cells (kidney collecting ducts, type-I alveolar lung cells, Purkinje cells of the cerebellum) [11]. The expression of the Duffy antigen on erythrocytes is linked to a single nucleotide polymorphism (SNP) (-33T>C) in the erythroid-specific promoter region (GATA box) of the DARC gene on chromosome 1 [12]. In carriers of the homozygous variant (-33CC) the Duffy antigen on the erythrocytes is absent (Duffy-negative phenotype), while in heterozygous carriers (-33TC) its expression is reduced [12, 13]. The Duffy-negative phenotype reaches frequency of 95–100% in West and Central African populations (and their descendants) and is extremely rare outside Africa [14], possibly explaining the low circulation of *P. vivax* in these geographical areas [1]. However, there is a growing number of reported cases of *P. vivax* infection in individuals with Duffy-negative erythroid phenotype, leading to hypothesize the existence of an alternative pathway for *P. vivax* erythrocyte invasion [12, 13, 15].

In Cameroon, a country of the Central Africa sub-region, *P. falciparum* is broadly considered responsible of up to 100% of malaria cases [1], and the vast majority (95–99%) of the population has a Duffy-negative erythroid phenotype [14]. Very recently *P. vivax* infections have been reported in Cameroon among symptomatic [16, 17] and asymptomatic [18] Duffy-negative individuals from different regions (South, South-West, Littoral, East) in the southern part of the country. The primary objective of the present study was to evaluate the *P. vivax* circulation among febrile outpatients seeking medical care in Dschang, West region of Cameroon. A second objective was to explore the Duffy antigen genotype frequency among the study population.

## Methods

### Study area and sample collection

The study was carried out in Dschang, chief town of the Menoua Division, West region of Cameroon. The city of Dschang (5°27′N; 10°04′E), which is located at an altitude of 1400 masl, has an average annual temperature of 20.5 ± 6 °C, with February being the hottest month. Four seasons can be distinguished as follows: the main dry season (November to mid-March), the short rainy season (mid-March to May), the short dry season (June to July) and the main rainy season (August to October) [19]. According to local health authorities, the 2012 estimated district’s population was of 218,006 inhabitants (17% under-5 years old children), with the vast majority belonging to Bamiléké, a Bantu-related ethnic group. The local economy is mainly based on agriculture, livestock and trade. The presence of non-African people in Dschang is very limited, and foreign tourism is almost absent.

Samples were collected consecutively from febrile outpatients (all native Cameroonian) attending the District Hospital of Dschang, West Cameroon, during a 3-months period (December to February). Demographic and essential clinical data were collected from each patient. After venepuncture, an aliquot of 100 µl of whole venous blood was spotted on filter paper (Whatman, UK), air-dried at room temperature, and then stored in locked bags at +4 °C.

### Nucleic acid extraction, analysis and sequencing reactions

Human/parasite DNA was extracted by automated method using Maxwell^®^16 instrument (Promega, Madison, WI, USA) from the dried blood spots (DBS) collected. The presence of nucleic acids or PCR inhibitors in the extracted DBS was determined by amplification of a human house-keeping gene (acid phosphatase 1) [20]. The PCR analysis of the four mains species of *Plasmodium* (*P. falciparum, P. vivax, Plasmodium ovale, Plasmodium malariae*) was performed by a nested-PCR of specific 18S rRNA gene fragments as previously described [21]. Four positive control DNA for all *Plasmodium* species and two negative (for the outer and nested PCR, respectively) were used in the genetic analysis [22].

**Fig. 1:**
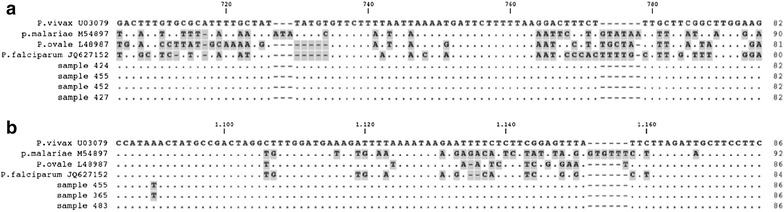
*Plasmodium vivax* (**a**) and *Plasmodium* spp. (**b**) sequence alignment* DNA sequence alignment of the four reference species (*P. vivax*, an. U03079; *P. malariae*, an. M54897; *P. ovale* an. L48987; *P. falciparum*, an. JQ627152) with four *P. vivax* positive amplicons (n. 424, 455, 452, 457) sequenced (**A**) and three *Plasmodium* spp. positive samples (n. 455, 365, 483) sequenced according to Rougemont et al. [26] (**B**). *Identical nucleotides are shown as *dots*, mismatches and gaps are highlighted in *gray*

To assess the specificity of the PCR analysis, four amplicons of *P. vivax* positive samples underwent sequencing reactions (Fig. 1) and they underwent to a further amplification of a region of the 18S rRNA gene different from the target region we used for the molecular detection [21]. Sequencing analyses were performed using an automated DNA sequencer (CEQ 8000, Beckman^®^). Sequencing alignments were carried out using ClustalX [23].

### Genotyping of erythrocytes Duffy antigen by melting curve analysis

**Fig. 2:**
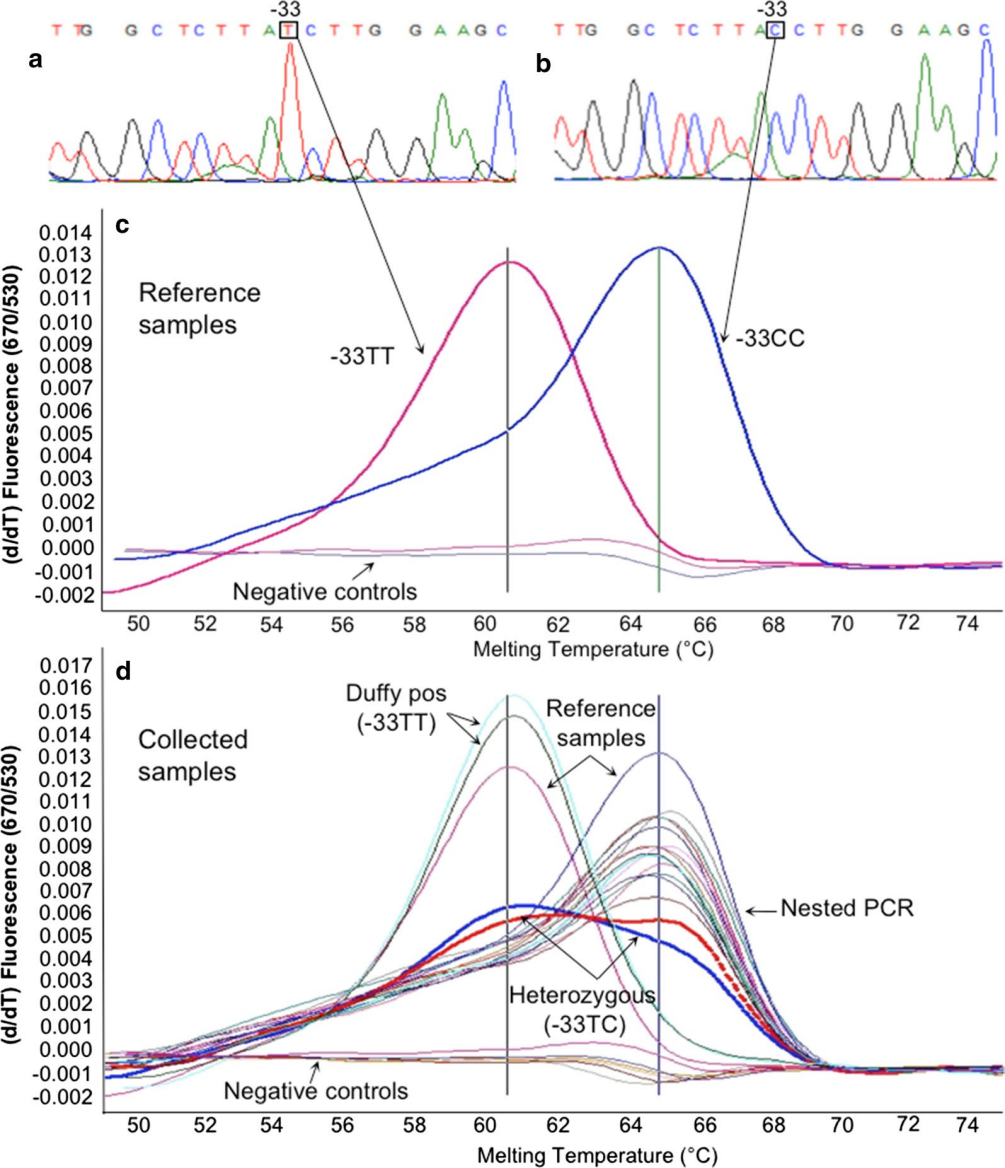
Duffy antigen genotyping (SNP -33T>C in GATA box, DARC gene) by melting curve analysis. **a** Chromatogram of the Caucasian Duffy-positive phenotype (-33TT genotype), reference sample. **b** Chromatogram of the Cameroonian Duffy-negative phenotype (-33CC genotype), reference sample. **c** Melting curve analysis of the two reference samples: melting temperatures of 60.5 and 64.5 °C for Duffy-positive (Caucasian) and Duffy-negative (Cameroonian), respectively. **d** Melting curve analysis of a representative group of samples, including the two reference samples. Two heterozygous samples (thick *red and blue lines*) are also shown. Negative controls are in the lower part of the melting curves of **c**, **d**

To explore the possible susceptibility of the study population to *P. vivax* infection, the Duffy antigen genotype due to the SNP -33T>C was investigated. Duffy genotype was analysed through the melting curve profile of a new-designed PCR method. The PCR amplified a fragment of 178 bp (from position 186–363, an. JN251917), encompassing the SNP in the GATA box (position 234). The sensor-probe was designed on the negative-phenotype. The PCR reaction was set-up in a final volume of 20 µl using the XtraTaq pol system [24] with 0.3 µM of forward (5′-CCTGTCCCTGCCCAGAA-3′) and 0.5 µM of reverse (5′-GGCATAGGGATAAGGGACT-3′) primers, 0.2 µM each of the anchor-probe (5′CY5-ACAGCCGTCCCAGCCC-3′PHO) and sensor probe (5′-TTACCTTGGAAGCACAGGCGC-3′FLU), 0.01% of BSA and 3 µl of DNA. The reactions were performed on a Light Cycler or LC480 instruments (Roche Diagnostics, Switzerland). The amplification program consisted of 95 °C for 30 s and then 45 cycles of 95 °C for 10 s, 52 °C for 30 s and 72 °C for 10 s. The transition rates (TR) were 20 °C/s. The melting curve analysis consisted of 95 °C for 30 s, 55 °C for 2 min with a TR of 20 °C/s, and an acquisition step from 50 to 75 °C with a TR of 0.1 °C/s. The fluorescence signal was acquired using channel 670/530. A melting curve analysis was performed on two homozygous controls showing two different peak profiles at 60.5 and 64.5 °C melting temperature, corresponding to -33TT (Caucasian, Duffy positive) and -33CC (Cameroonian, Duffy negative) genotypes, respectively (Fig. 2c). The positive controls were also sequenced (Fig. 2a, b).

### Statistical analysis

Categorical variables (sex, provenance, ethnicity, pregnancy, comorbidities, previous anti-malarial self-medication) were reported as absolute number and percentage, and continuous data (age, days of diagnostic delay) were expressed as median and interquartile range. Chi square test (for categorical variables) and Mann–Whitney test (for continuous variables) were used to compare *Plasmodium* spp. positive *vs* negative and *P. falciparum* positive vs *P. vivax* positive results. D’Agostino and Pearson omnibus normality test was applied to confirm the non-normal distribution of quantitative parameters. Odds ratio (OR) with 95% confidence interval (CI) were also calculated. The R (version 2.15.0, R foundation statistical Computing) and Prism (version 5.00.288, GraphPad Software, Inc) were employed as statistical software. Evaluation of Hardy–Weinberg equilibrium (HWE) was performed using the HWSIM software [25] and Monte-Carlo permutation test performed when genotypic classes had an expected cell size of less than five.

## Results

A total of 484 samples were consecutively collected from febrile outpatients. Population’s characteristics are summarized in Table 1. Malaria parasite DNA was identified by nested-PCR in 70 samples (14.5%): 68 cases of *Plasmodium* mono-infections (42 *P. falciparum*, 25 *P. vivax*, and 1 *P. malariae*) and 2 cases of *P. falciparum*/*P. vivax* co-infections. Because of the unexpected frequency of *P. vivax*, in order to assess the specificity of the molecular analysis, four amplicons were purified, sequenced and aligned confirming the specificity of the analysis (Fig. 1a). Additionally, a subset of *P. vivax* positive samples by molecular analysis was analyzed by amplifying a different region of the rRNA 18S gene [26]: three amplicons were sequenced and aligned confirming the *P. vivax* diagnosis (Fig. 1b). The proportion of *P. falciparum* and *P. vivax* infections was 9.1% (n = 44/484) and 5.6% (n = 27/484), respectively. Notably, 38.6% (n = 27/70) of *Plasmodium* positive PCR results were due to *P. vivax* infection. Male gender was associated with *Plasmodium* PCR positive result (OR 2.3; 95% CI 1.39–3.89; *P* = 0.0014) at univariate analysis (Table 1). No other correlation was found between PCR results (or *Plasmodium* species detected) and other demographic or clinical data collected (Table 1).

**Table 1:** Study population characteristics and comparison between *Plasmodium* spp. (*P.* spp.) PCR-pos vs PCR-neg groups, and between PCR-pos *P. falciparum* (*Pf*) vs *P. vivax* (*Pv*) patients (univariate analysis)

Variable	Total population	*P.* spp. PCR− (n = 414)	*P.* spp. PCR+ (n = 70)^a^	*P* value	*Pf* PCR+ (n = 42)^b^	*Pv* PCR+ (n = 25)^b^	*P* value
Age (years), n (%)							
≤5	131 (27.1%)	112 (27.1%)	19 (27.1%)	*0.153*	11 (26.2%)	7 (28.0%)	*0.906*
6–14	31 (6.4%)	26 (6.3%)	5 (7.1%)		2 (4.8%)	2 (8%)	
15–24	91 (18.8%)	78 (18.8%)	13 (18.6%)		9 (21.4%)	4 (16%)	
25–64	198 (40.9)	171 (41.3%)	27 (38.6%)		17 (40.5%)	9 (36%)	
≥65	33 (6.8%)	27 (6.5%)	6 (8.6%)		3 (7.1%)	3 (12%)	
Median [IQR]	24 [4–40]	23 [4–43]	24 [4–39]	*0.732*	23 [4–42]	21 [3–37]	*0.721*
Sex, n (%)							
Male	191 (39.5%)	151 (36.5%)	40 (57.1%)	*0.001*	24 (57.1%)	14 (56%)	*1.000*
Female	293 (60.5%)	263 (63.5%)	30 (42.9%)		18 (42.9%)	11 (44%)	
Provenance, n (%)							
Urban	316 (65.3%)	264 (63.8%)	52 (74.3%)	*0.103*	30 (71.4%)	19 (76%)	*0.780*
Rural	168 (34.7%)	150 (36.2%)	18 (25.7%)		12 (28.6%)	6 (24%)	
Ethnicity, n (%)							
Bamiléké	429 (89%)	363 (88%)	66 (94%)	*0.151*	40 (95%)	24 (96%)	*1.000*
Others	55 (11%)	51 (12%)	4 (6%)		2 (5%)	1 (4%)	
Co-morbidity, n (%)							
No	448 (92.6%)	383 (92.5%)	65 (92.9%)	*1.000*	38 (90.5%)	24 (96%)	*0.643*
Yes	36 (7.4%)	31 (7.5%)	5 (7.1%)		4 (9.5%)	1 (4)	
Diabetes	8 (1.6%)	8 (1.9%)	0 (0%)		0 (0%)	0 (0%)	
AHT	19 (3.9%)	15 (3.7%)	4 (5.7%)		3 (7.1%)	1 (4%)	
AHT + diabetes	5 (1.1%)	5 (1.2%)	0 (0%)		0 (0%)	0 (0%)	
AHT + bronchial asthma	1 (0.2%)	1 (0.2%)	0 (0%)		0 (0%)	0 (0%)	
Bronchial asthma	1 (0.2%)	1 (0.2%)	0 (0%)		0 (0%)	0 (0%)	
HIV infection	1 (0.2%)	1 (0.2%)	0 (0%)		0 (0%)	0 (0%)	
HCV infection	1 (0.2%)	0 (0%)	1 (1.4%)		1 (2.4%)	0 (0%)	
Pregnancy, n (%)^c^							
Yes	17 (5.8%)	15 (5.7%)	2 (6.7%)	*0.688*	0 (0%)	2 (18.2%)	*0.135*
No	276 (94.2%)	248 (94.3%)	28 (93.3%)		18 (100%)	9 (81.8%)	
Previous anti-malarial self-medication, n (%)^d^							
Yes	198 (40.9%)	168 (40.6%)	30 (42.9%)	*0.952*	18 (42.9%)	11 (44%)	*0.952*
No	286 (59.1%)	246 (59.4%)	40 (57.1%)		24 (57.1%)	14 (56%)	
Diagnostic delay (days)							
Median [range]	3 [2–7]	3 [1–6]	3 [2–7]	*0.144*	3.5 [2–7]	3 [2–7]	*0.707*

*[IQR]* interquartile range, *AHT* arterial hypertension

^a^68 *Plasmodium* mono-infections (42 *P. falciparum*, 25 *P. vivax*, 1 *P. malariae*) and 2 *P. falciparum* + *P. vivax* co-infections

^b^Two co-infected (*P. falciparum* + *P. vivax*) patients not included in the analysis

^c^Only among women

^d^Anti-malarial drugs taken after the onset of fever and before the medical consultation

The Duffy antigen genotype (Fig. 2; Table 2) was assessed in a subset of 228 unrelated samples (27 *P. vivax* positive and 201 randomly selected). Two Duffy-positive (-33TT), two heterozygous (-33TC), and 224 Duffy-negative (-33CC) genotypes were detected. All *P. vivax* infected individuals showed a Duffy-negative genotype. Overall, the frequency of the -33T allele was 1.3%, corresponding to a frequency of 1.7% (n = 4/228) of Duffy-positive phenotypes (homo- and heterozygotes). Then the HWE was tested. A lack of heterozygotes was assessed using Chi square analysis (χ^2^ = 99.99, 1 df, *P* < 0.001). The Monte-Carlo permutation test (10,000 iterations) gave a *P* = 0.0629.

**Table 2:** Duffy-antigen genotyping results (n = 228)

PCR-analysis results	Duffy-antigen genotyping		
	-33TT	-33TC	-33CC
*P. falciparum* positive	0	0	11
*P. malariae* positive	0	0	1
*P. vivax* positive	0	0	25
*P. falciparum/P. vivax* positive (co-infection)	0	0	2
*Plasmodium* negative	2	2	185
Total	2	2	224

## Discussion

The evidence of *P. vivax* infection in Duffy negative febrile individuals from the West Region of Cameroon is reported. The study population was mainly represented by young individuals (median age 24 years), and in particular children. Notably, the only factor associated with a higher frequency of malaria infection was male gender (see Table 1): a possible explanation of this statistically significant difference could be that male patients seek for medical advice only in more severe cases, or due to chance alone.

**Fig. 3:**
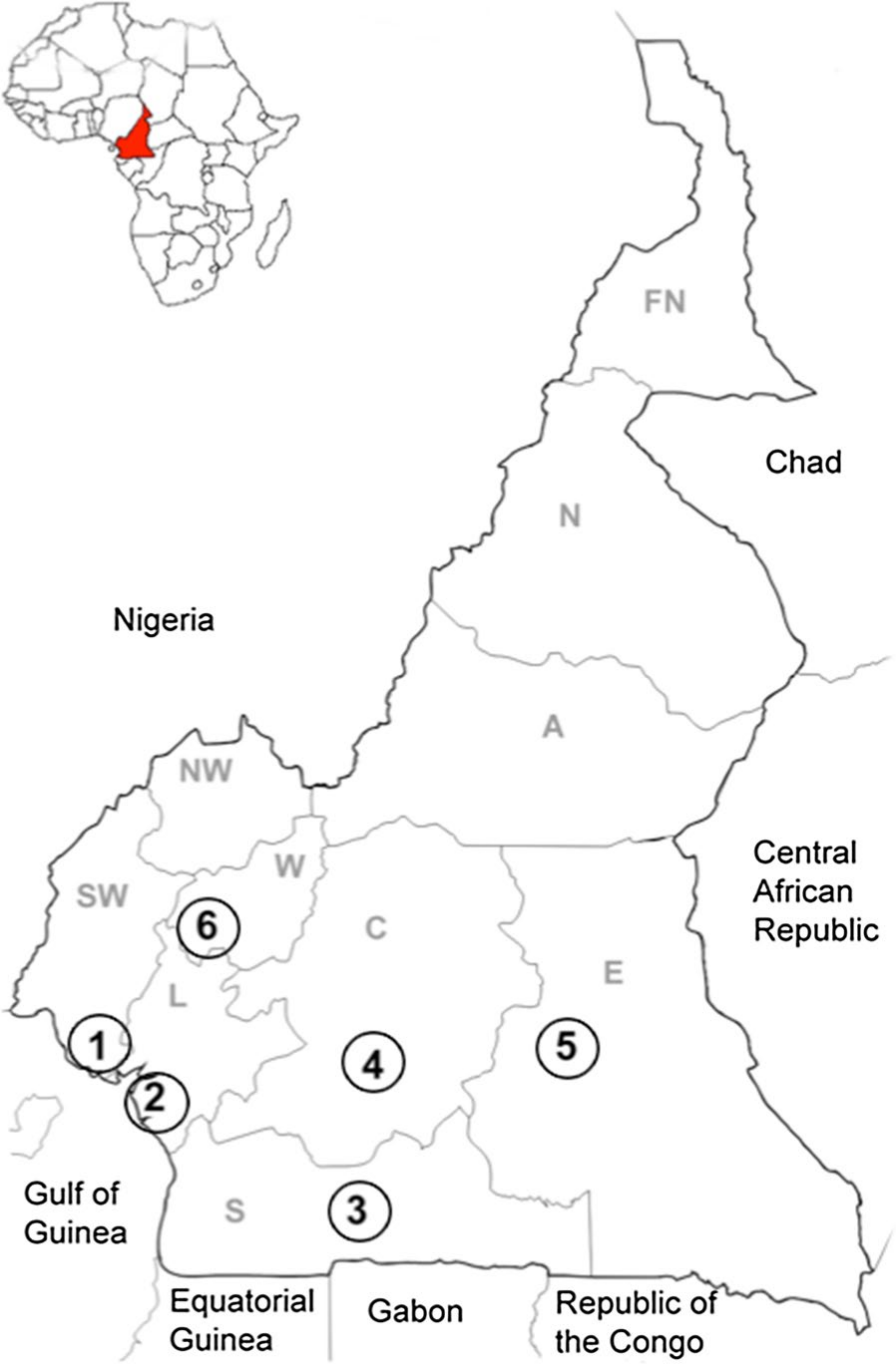
*Plasmodium vivax* infection in Cameroon. *Cameroonian regions: FN* Far North, *N* North, *A* Adamaoua, *NW* North-West, *SW* South-West, *W* West, *L* Littoral, *C* Centre, *S* South, *E* East. *Study sites*: *1* Bolifamba village (South-West Region): 13/269 (4.8%) *P. vivax* infections [18]; *2* Douala (Littoral Region): 2/52 (3.8%) [16] and 6/60 (10%) [17] *P. vivax* infections; *3* Ebolowa (South Region): 3/60 (5%) *P. vivax* infections [16]; *4* Yaoundé (Centre Region): 1/29 (3.4%) *P. vivax* infection [16]; *5* Bertoua (East region): 2/25 (8%) *P. vivax* infections [16]; *6* Dschang (West Region): 27/484 (5.6%) *P. vivax* infections (present study)

Historically, *P. vivax* infection has been considered as “benign” malaria, and the Duffy-negative phenotype as full protective against *P. vivax* infection. But, recently, both axioms have been questioned. In fact, there are several reports of severe vivax malaria [4, 8, 9] as well as of *P. vivax* infection among Duffy-negative individuals from Angola [27], Cameroon [16–18], Equatorial Guinea [27], Ethiopia [28, 29], Kenya [30], Madagascar [13], Mauritania [31] and Brazil [32, 33]. Three recent studies showed the circulation of *P. vivax* in the southern part of Cameroon (Table 3; Fig. 3) [16–18]. In comparison with these studies [16–18] data from the West region of Cameroon confirm the circulation of *P. vivax* in native individuals with Duffy-negative genotype (Fig. 3), and showed a higher relative proportion of *P. vivax* infection, possibly linked to the higher altitude of the study area. Moreover, considering that the study was conducted when the anopheline density was at its lowest (main dry season) [19], these data possibly underestimate the real circulation of *P. vivax* in West-Cameroon, where climatic conditions during the two rainy seasons may facilitate its circulation [2, 3].

**Table 3:** Vivax malaria in Cameroon

Study (ref.)	Study site	Total population, n	Malaria cases, n (%)	Vivax malaria cases		
				n	%^a^	Duffy neg, n
[16]	Five sites^b^	485^c^	201 (41.4)	8	4	8/8
[17]	Douala city	60^c^	43 (71.7)	10	23.2	10/10
[18]	Bolifamba village^d^	269^e^	87 (32.3)	13	4.8	6/13
Present study	Dschang^f^	484^c^	70 (14.5)	27	38.6	27/27

^a^Relative proportion of *P. vivax* infection among total malaria cases

^b^Five sites in southern Cameroon (Littoral, South, Centre and East regions)

^c^All symptomatic (febrile) individuals

^d^South-West region of Cameroon

^e^All asymptomatic individuals

^f^West region of Cameroon

The Cameroonian population, as well as other West and Central African populations (and their descendants), show a 95–99% frequency of Duffy-negative phenotype [14], possibly as a consequence of the positive selective process linked to an ancient (100,000 years) presence of *P. vivax* in sub-Saharan Africa [3]. In this study, the HWE assessment on the Duffy genotype distribution showed a lack of heterozygotes (using Chi square analysis), and the Monte-Carlo permutation test being on the verge of significance. It is noteworthy that the slight departure from the HWE of genotype frequencies for Duffy is probably due to the effect of natural selection exerted by *P. vivax* (one of the theoretical assumptions for HWE is absence of natural selection), similar to that seen in Madagascar [34]. It is also worth considering the possible deviation from HWE with respect to the biased sampling of febrile individuals.

According to some authors, the maintenance of *P. vivax* circulation in Duffy-negative population context is possibly related to the presence of reservoirs of Duffy-positive individuals (1–5%) presumably present in the local population [5] or, in restricted areas, to *P. vivax* and/or *P. vivax*-like infections found in apes [35]. Moreover, *P. vivax* circulation might be facilitated by the highly specific vectorial competence showed by the *Anopheles gambiae* and *Anopheles funestus* complexes that circulate in Africa [30, 36]. In this study, the measure Duffy antigen expression on erythrocytes at the protein level was not assessed; however, the Duffy genotype predicts its phenotype with high consistency [13]. In the population study, the -33T allele frequency was 1.3%, confirming a minimal presence of Duffy-positive individuals also within the Bamiléké ethnic group in West Cameroon. Moreover, the country (mainly in the southern part) accounts for a significant population of great apes; a recent study has showed a relevant 2.3% (n = 45/2168) of *P. vivax*-like infection among great apes living in Cameroon [37]. These elements, together with the climate and geographic variety of Cameroon, suggest that *P. vivax* circulates in different areas of the country. Some investigators suggest that *P. vivax* may be in the process to evolving the ability to infect Duffy-negative erythrocytes [13, 15], possibly involving parasite DBPs and/or reticulocyte binding proteins [38, 39]. The alternative yet-uncharacterized Duffy-independent erythrocyte invasion pathway is likely to be less efficient [13], leading to lower level of parasitaemia as observed in carriers of heterozygous Duffy-genotype (-33TC) from Papa New Guinea [40]. Thus, the reason underlying the reported *P. vivax* infection in Duffy-negative individuals from context with very low presence of Duffy-positive phenotypes, as in the present study, remains unclear. All these elements underline the basic knowledge gaps of *P. vivax* life cycle [7–9] and the need to assess the real circulation of *P. vivax* in sub-Saharan Africa [41] also in the broader perspective of malaria eradication worldwide.

Although *P. vivax* is susceptible to several anti-malarial drugs (i.e. chloroquine, quinine, artemisinin and its derivatives), primaquine (PQ) is the only licensed drug active against the hypnozoites to prevent *P. vivax* relapses from the liver [6]. Moreover, a true resistance to PQ in *P. vivax* hypnozoites has not been described, suggesting a role for host factors in drug failure [42]. Primaquine is metabolized in the liver by the enzyme cytochrome P450-2D6 (CYP2D6), possibly leading to a metabolite responsible for hypnozoite killing [42]. Thus, defective CYP2D6 metabolism could be associated with PQ failure [43]. Furthermore, the risk of acute haemolytic anaemia in carriers of glucose-6-phosphate dehydrogenase (G6PD) deficiency is a safety concern for PQ use (14 days at 0.5 mg/kg), and data on African G6PD deficiency are not exhaustive [44]. Available data related to G6PD in Cameroon are scarce, with one published survey reporting the G6PD deficiency prevalence being 6.6% [45]. Thus, considering the observed *P. vivax* circulation in different regions of Cameroon, larger studies assessing G6PD deficiency prevalence and *CYP2D6* polymorphism frequency in the whole country are necessary in order to ensure a safer therapeutic use of PQ.

The present study has some limitations. Data on parasitaemia of *Plasmodium* species infections are lacking because the diagnosis was based only on a molecular qualitative technique and because of the lack of experienced local microscopists. Moreover, although 40% of the participants reported anti-malarial self-medication before the medical consultation, data on drugs, dosage and duration were not reported, as well as follow-up data of infected cases.

## Conclusion

The present study reports the molecular evidence of *P. vivax* circulation in the West region of Cameroon among symptomatic Duffy-negative outpatients, perhaps with the highest proportion of *P. vivax* infection when compared to previous studies in the country [16–18]. These results suggest the need for assessing the real circulation of *P. vivax* in West/Central African countries to plan public health activities in order to improve the local microscopic diagnostic capacity, and to ensure a more effective and safer therapeutic management of vivax malaria attacks and relapses.
